# Intestine-Specific, Oral Delivery of Captopril/Montmorillonite: Formulation and Release Kinetics

**DOI:** 10.1007/s11671-010-9749-0

**Published:** 2010-08-27

**Authors:** Suguna Lakshmi Madurai, Stella Winnarasi Joseph, Asit Baran Mandal, John Tsibouklis, Boreddy SR Reddy

**Affiliations:** 1 Industrial Chemistry Laboratory, Central Leather Research Institute, Council of Scientific and Industrial Research, Chennai 600 020, India; 2 Biomaterials & Drug Delivery Research Group, School of Pharmacy and Biomedical Sciences, University of Portsmouth, Portsmouth, Hampshire PO1 2DT, UK

**Keywords:** Captopril montmorillonite, Intercalation, Intestine-specific controlled release, Release kinetics

## Abstract

The intercalation of captopril (CP) into the interlayers of montmorillonite (MMT) affords an intestine-selective drug delivery system that has a captopril-loading capacity of up to *ca*. 14 %w/w and which exhibits near-zero-order release kinetics.

## Introduction

Captopril (CP; 1-[(2s)-3-mercapto-2-methyl propionyl]-L- proline), an orally active inhibitor of angiotensin-converting enzyme (ACE) [1,2], is in many countries the medication of choice for the management of hypertension and is often used to treat some types of congestive heart failure [3-6]. CP contains a reactive thiol group, which is postulated to bind to the Zn^2+^ of the angiotensin-converting enzyme [7] and which forms the disulfide linkages with thiol-containing residues of plasma proteins that are responsible for the extensive tissue binding of the drug [8]. Owing to its pKa (3.7 at 25°C), CP is highly soluble in water at acidic pH (125–160 mg/ml at pH 1.9). At pH > pKa, the amidic linkage of the molecule becomes increasingly susceptible to hydrolysis; under basic conditions, the drug exhibits a pseudo-first-order degradation reaction [9,10].

In man, CP reduces plasma angiotensin II and aldosterone levels, increases plasma renin activity and produces a significant decrease in blood pressure in hypertensive patients [11]. It blocks the enzyme system that causes the relaxation of artery walls, reducing blood pressure, decreasing symptoms of cystinuria and reducing rheumatoid arthritis symptoms. The duration of the antihypertensive action of a single oral dosing of CP is 6–8 h, with the implication that clinical administration requires the daily dose of 37.5–75.0 mg to be taken at 8-h intervals [12]. The metabolic products of CP include a disulfide dimer of CP, a CP-cysteine disulfide and mixed disulfides with endogenous thio compounds [13]. In efforts to reduce the frequency of administration, several attempts have been made to design sustained release formulations. These have included coated tablets [14-16], beadlets [17], hydrophobic tablets [18], pulsatile delivery systems [19], microcapsules [20], semisolid matrix systems [9], floating tablets and capsules [21], and bioadhesive polymers [22].

An evolving approach to controlled drug delivery involves the use of nanoclays with well-defined morphologies. Montmorillonite (MMT), a swelling clay mineral, is one such material that has shown considerable promise as a carrier in controlled drug delivery. Since the mineral is comprised of alternating negatively charged alumino-silicate layers with exchangeable counter ions positioned between each layer [23], the capability of the material to act as a controlled delivery vehicle is rationalized in terms of the potential for drug molecules to become adsorbed onto the hydrated alumino-silicate layers, which in aqueous media exist as dispersions of individual platelet. This paper describes an attempt to assess the suitability of MMT to act as a matrix for the controlled release of CP by evaluating intercalation data from three methods (solution, melt and grinding) and by considering the characteristics of CP release.

## Materials and Methods

### Materials

**Figure 1:**
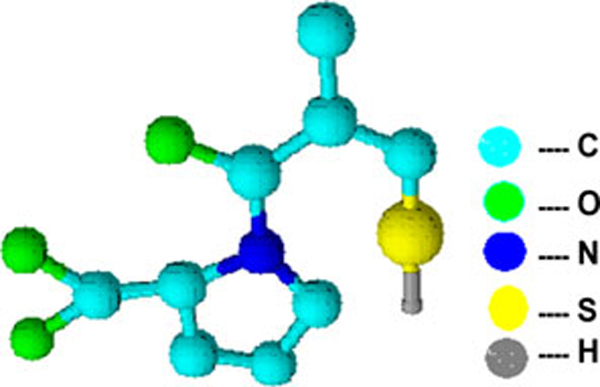
**Structure of CP**.

K10 Montmorillonite nanoclay (specific surface area = 274 m^2^/g, cation exchange capacity = 119 Meq/100 g) was purchased from Sigma–Aldrich, USA. Captopril (Figure 1; melting point 106°C) was sourced from Medrich pharmaceuticals, India, and was used as received. All the other chemicals used were of analytical grade.

### Preparation of CP-MMT Systems

**Figure 2:**
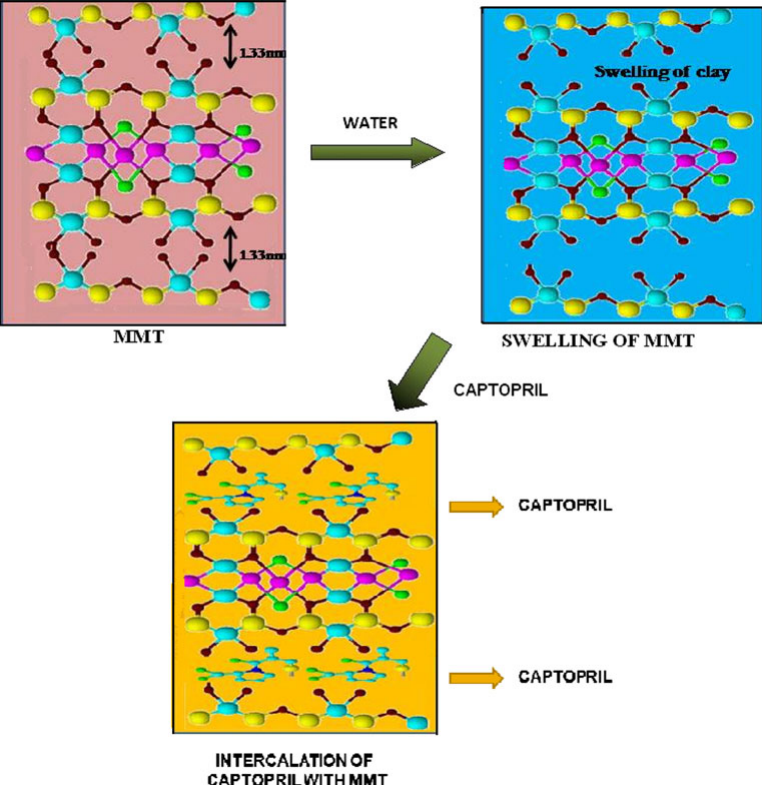
**Schematic representation of intercalation of CP into MMT**.

Three methods (solution, melt and grinding) were employed for the intercalation of CP into the MMT matrix (Figure 2, schematic representation of intercalation process).

### Optimization of Clay Colloidal Dispersion

**Figure 3:**
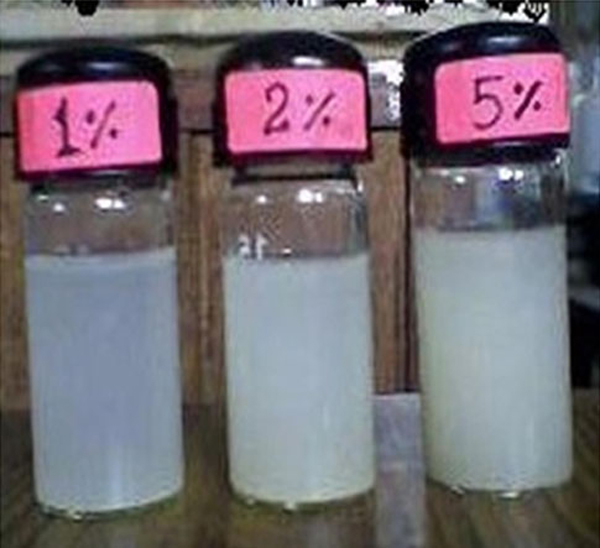
**Colloidal dispersions**.

Accurately weighed amounts of MMT nanoclay (*ca*. 1, 2 or 5 g) were dispersed separately in vessels containing deionized water (100 ml) and allowed to stand for about 15 h and stirred (magnetic stirrer) for 24 h. The colloidal stability of the dispersions was assessed visually over 24 h. Since all dispersions appeared stable within this timescale, the more concentrated, 5 %w/w MMT, dispersion was selected for further evaluation (Figure 3).

### Solution Intercalation Method

To improve the cation exchange capacity (CEC) of the clay, MMT-K10 was treated with sodium chloride and the resultant Na-MMT dispersions were washed with deionised water (centrifugation) until a AgNO_3_ test confirmed that all chloride had been removed [24]. CP (1.382, 2.765, 3.456 and 4.417 mM) was added to separate vessels containing the 5 %w/w Na-MMT aqueous dispersion (100 cm^3^) and maintained (stirring) at 50°C for 4 h. To remove any free drug, the intercalated particles were collected following repeated (4×; replacing the deionized water after each cycle) centrifugation (4,000 rpm, 20 min) of the dispersion. The isolated CP-MMT powder was dried in a vacuum oven, ground and stored in a desiccator. To assess the improvement in cation exchange capacity following treatment with sodium, samples of MMT were subjected to an identical procedure and used as controls.

### Melt Intercalation Method

A mixture of MMT and CP (10:9 w/w) was heated (2°C/min) to the melting point of CP and maintained at that temperature for 6 h. The cooled (room temperature) residue was washed (3×) with deionised water and dried (room temperature) before use.

### Grinding Intercalation Method

A mixture of MMT and CP (10:9 w/w) was ground finely (*ca*. 30 min) using a pestle and mortar, washed (deionised water, 3×) and dried (desiccator) before use.

### *In Vitro* Drug Release

The simulated gastric fluid was a buffer solution (pH 1.2) that had been prepared by mixing 250 ml of aqueous HCl (0.2 M) with 147 ml of aqueous KCl (0.2 M). The simulated intestinal fluid was a buffer solution (pH 7.4) that had been prepared by mixing 250 ml of aqueous KH_2_PO_4_ (0.1 M) and 195.5 ml of aqueous NaOH (0.1 M) [25].

The drug release study was performed in a constant temperature bath (37°C) fitted with a rotating round-bottomed flask (100 rpm) by suspending a dialysis membrane bag containing 20 ml of CP-MMT dispersion in 900 ml of dissolution media. At specified time intervals, an aliquot (5 ml) of the dissolution medium was removed and the concentration of CP was determined by UV absorption measurements, respectively, at 205 and 217 nm for the acidic and basic buffers.

### Drug Release Kinetics

To assess the kinetics of CP release, in vitro drug release data were fitted into established mathematical models.

To assess zero-order release kinetics, the relationship between the rate of drug release and its concentration was examined from a plot of percentage drug release *vs*. time:

(1) $$Q_{t}=Q_{o}+K_{o}t$$

where, *Q*_*o*_ = initial amount of drug, *Q*_*t*_ = cumulative amount of drug release at time *t*, *K*_*o*_ = zero-order rate constant and *t* = time in h.

A log plot of percent drug remaining *vs*. time allowed the assessment of first-order kinetics.

(2) $$\log Q_{t}=\log Q_{o}+K_{1}t/2.303$$

where, *K*_1_ = first-order rate constant.

Fickian diffusion was assessed using the Higuchi model, which plots percentage drug release against the square root of time.

(3) $$Q=K_{H}t^{1/2}$$

where, *Q* = cumulative drug release at time *t* and *K*_*H*_ = constant reflective of the design variables of the system.

Additionally, the Korsmeyer–Peppas model, which has been designed to identify the release mechanism of a drug/drug carrier system, was employed to assess data collected during the first 210 min of the in vitro experiment.

(4) Mt/M∞=Ktn

Where, *Mt/M∞* = fraction of drug released at time *t*, *K* = rate constant and *n* = release exponent.

Values of *n* between 0.5 and 1.0 are indicative of anomalous, non-Fickian, kinetics [26].

### Characterization

The concentration of CP was determined from calibration plots of absorbance (SHIMADZU UV 240 Spectrophotometer; quartz cell path length = 1 cm) at 205 nm or at 217 nm for the molecule in acidic or alkaline buffer, respectively. Infrared spectra (KBr disks) were recorded using a PERKIN-ELMER Spectrum RX1, FTIR V.2.00 spectrophotometer. X-ray diffraction (XRD) patterns were recorded using a SIEMENS D-500 variable angle diffractometer (CuKα source, λ = 1.5405 A°; 1–60°). Thermogravimetric determinations (37–800°C, 10°C/min; TA instruments TGA Q50) were carried out under nitrogen.

## Results and Discussion

### CP-MMT Intercalation

**Figure 4:**
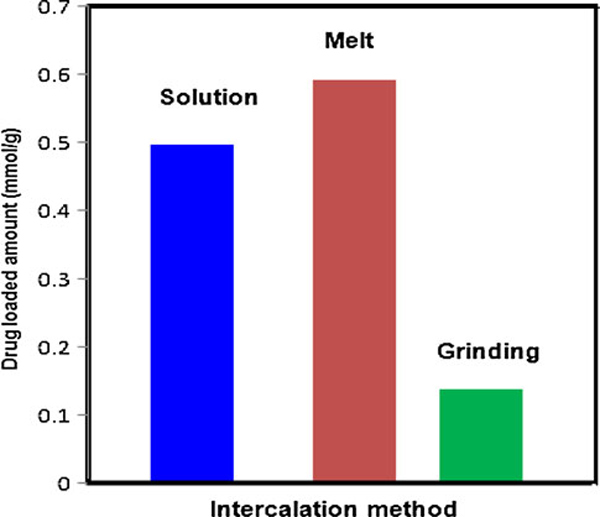
**Drug-loading capacities of CP-MMT systems prepared by solution, melt and grinding intercalation**.

The drug-loading capacities for CP-MMT systems that had been formed by the solution, melt and grinding methods are presented in Figure 4. In accord with the susceptibility of CP (pKa = 3.7) to hydrolytic degradation, solution intercalation was performed in acidic media. The CP-loading capacity of Na-MMT was very similar to that of MMT-K_10_.

### FT-IR Analysis

**Figure 5:**
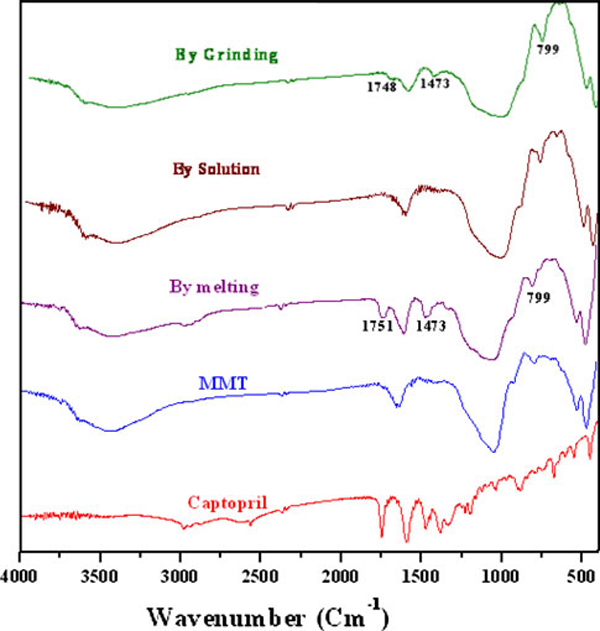
**FT-IR spectra of CP, MMT and of CP-MMT systems**.

In Figure 5 are presented the infrared spectra of pure MMT, pure CP and CP-MMT composites that had been prepared using the solution, melt or grinding methods. The spectrum of pure MMT is characterized by the stretching and bending vibrations of Si–O–Si and Si–O–Al, correspondingly at 1,048 cm^-1^ and 528.57 cm^-1^, and by the 919 cm^-1^ stretch of Al–Al–OH moieties in the octahedral layer. Interlayer water is manifest by the broad –O–H stretching band at *ca*. 3,400 cm^-1^. The bands at 3,623 cm^-1^ and at 3,698 cm^-1^ are respectively attributed to the –OH stretch of Al–OH and that of Si–OH [25]. The –OH bending mode of absorbed water is evidenced as a series of overlapping bands at 1,661 cm^-1^. In the spectrum of pure CP, the C=O stretching mode, amide absorption, S–H stretch and C–S stretch are respectively seen at 1,751 cm^-1^, 1,587 cm^-1^, 2,570 cm^-1^ and 678 cm^-1^. The spectra of the CP-MMT systems were dominated by the features of MMT, but there was considerable variation in the shape, position and relative intensity of individual spectral features. The band at 1,751 cm^-1^, which is absent in the spectrum of MMT but features strongly in that of CP, is interpreted as evidence for CP-MMT intercalation.

### XRD Analysis

**Figure 6:**
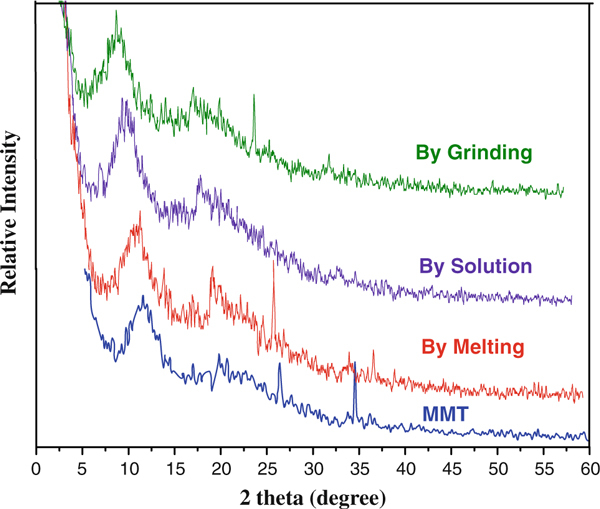
**XRD patterns for MMT and for CP-MMT systems**.

Comparison of the XRD pattern of pure MMT with those of CP-MMT composites from solution, melt or grinding methods (Figure 6) confirms that the clay retains its structure following intercalation. Consistent with previous reports that the method of intercalation impacts upon the d-spacing of the carrier mineral [27,28], the characteristic (001) peak of pure MMT (2[*θ*] = 9.9°) shifts to 11, 11.5 and 9.6°, respectively, for CP-MMT composites prepared by solution, melt or grinding methods. The interlayer distances CP-MMT systems prepared by solution, melt and grinding methods were characterized by respective basal spacing values of 1.7, 2.4 and 1.6 nm (Table 1). Since the corresponding distance for MMT is 1.3 nm, the more open structure at the (001) plane of CP-MMT composites is interpreted as evidence for the successful intercalation of CP into the interlayer structure of the mineral.

**Table 1:** Basal spacings of CP-MMT systems, as determined by XRD

Intercalation method	Drug loaded amount (mmol/g)	Interlayer distance (nm)
MMT	–	1.3
CP-MMT by solution	0.498	1.7
CP-MMT melt	0.593	2.4
CP-MMT grinding	0.137	1.6

### Thermogravimetric Analysis

**Figure 7:**
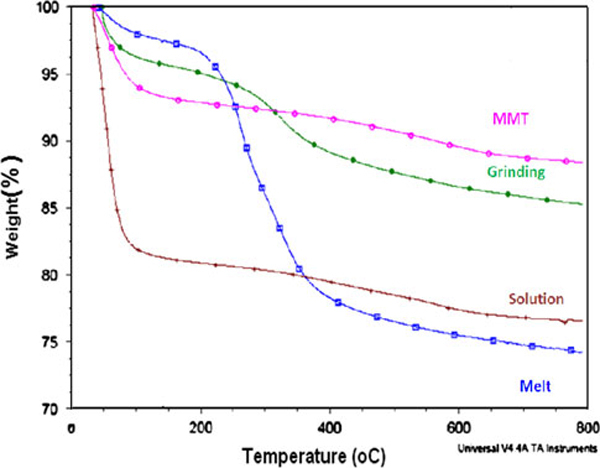
**Thermograms of CP-MMT systems**.

The thermogram of MMT is characterized by a 7% mass loss, which at the heating rate of 10°C/min occurred over the temperature range of 48–120°C and is consistent with the desorption of water molecules from MMT. The thermograms of CP-MMT systems are characterized by the decomposition of intercalated CP (200–250°C) and by a second mass loss of 6% (430–450°C), which corresponds to the structural dehydroxylation of MMT, Figure 7.

### CP Release Profiles

**Figure 8:**
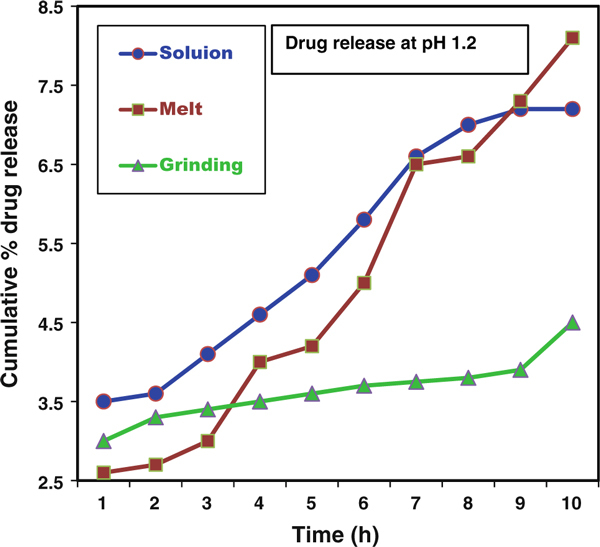
**Drug release patterns of CP-MMT systems at pH = 1.2**.

**Figure 9:**
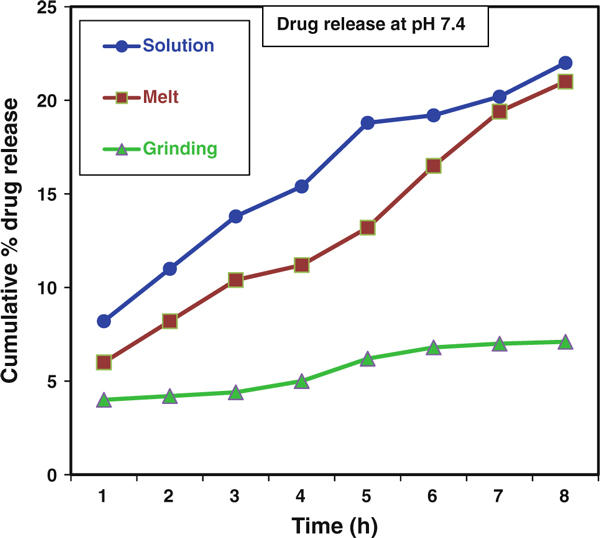
**Drug release patterns of CP-MMT systems at pH = 7.4**.

The controlled release patterns and pH dependences of the rate of CP release from each of the CP-MMT matrixes are illustrated by the cumulative drug release data presented in Figures 8 and 9. In intestinal-fluid-mimicking medium (pH 7.4), CP release over 9 h was 22, 21 and 4%, respectively, for CP-MMT prepared by the melt, solution and grinding methods, Table 2. Corresponding values for the gastric-fluid-mimicking medium (pH 1.2) were considerably lower, indicating the potential of the formulation to exhibit small-intestine selectivity.

**Table 2:** Drug release profiles of CP-MMT systems

Intercalation method	Drugloaded amount (mmol/g of clay)	Drug release rate (%) at	
		
		pH 1.2	pH 7.4
Solution	0.498	7.2	22.0
Melt	0.593	8.1	21.0
Grinding	0.137	4.5	7.1

### Drug Release Kinetics

**Figure 10:**
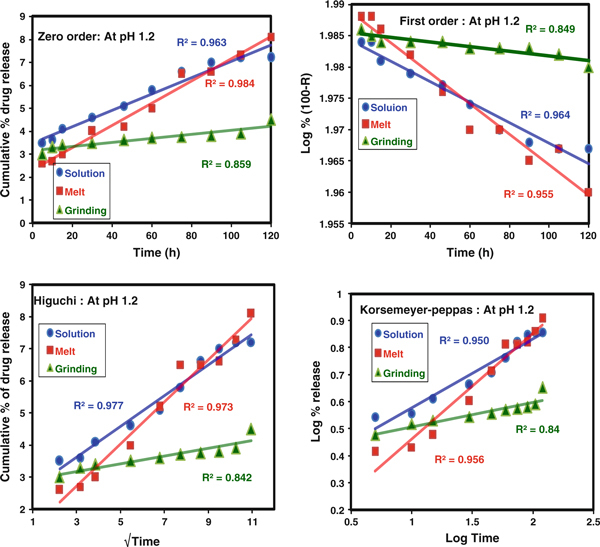
**Zero order, First order, Higuchi and Koresmeyer–Peppas kinetic models at pH 1.2**.

**Figure 11:**
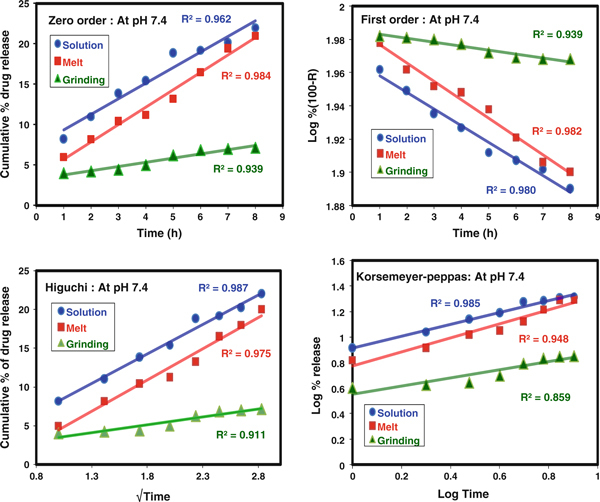
**Zero order, First order, Higuchi and Koresmeyer–Peppas kinetic models at pH 7.4**.

Fitting of the data, from the in vitro release of CP from the CP-MMT matrix, to the theoretical models (Figures 10 and 11) showed that, at both pH values considered, the release profiles of formulations prepared in the melt or by grinding were consistent with near-zero-order kinetics. Comparison of the correlation coefficients (R^2^, Tables 3 and 4) identified the Higuchi model as that which fits the data best, irrespective of the pH of the release medium. In all the cases, values of *n* < 0.5 indicated that the drug diffusion mechanism is classical, non-Fickian release, which is assumed to be facilitated by the swelling of the clay matrix [29]. The application of the Korsmeyer–Peppas model was consistent with the suitability of the CP-MMT system to act as an orally administered vehicle for the sustained release of CP [30].

**Table 3:** Parameters for CP release at pH 1.2

pH 1.2	Zero order	First order	Higuchi	Koresmeyer–Peppas	
				
	**R**^**2**^	**R**^**2**^	**R**^**2**^	**R**^**2**^	*n*
Solution	0.963	0.964	0.977	0.950	0.255
Melt	0.984	0.955	0.973	0.956	0.391
Grind	0.859	0.849	0.842	0.840	0.091

**Table 4:** Parameters for CP release at pH 7.4

pH 7.4	Zero order	First order	Higuchi	Koresmeyer–Peppas	
				
	**R**^**2**^	**R**^**2**^	**R**^**2**^	**R**^**2**^	*n*
Solution	0.962	0.980	0.987	0.985	0.466
Melt	0.984	0.982	0.975	0.948	0.551
Grind	0.939	0.939	0.911	0.859	0.321

## Conclusions

CP has been confirmed to successfully intercalate into the interlayers of MMT. The maximum percentage of intercalated CP was determined as *ca*. 14 %w/w. In vitro release experiments have shown that the release of CP from the MMT matrix is sensitive to the pH of the dissolution media. The CP release rate in simulated intestinal fluid (pH 7.4) is significantly higher than that in simulated gastric fluid (pH 1.2) and exhibits near-zero-order release kinetics.

## References

[B1] Ferguson RK Brunner HR Turini GA Gavras H McKinstry DN Lancet 1977 1 775 10.1016/S0140-6736(77)92958-0 66571 66571

[B2] Ondetti MA Rubin B Cushman DW Science 1977 196 441 10.1126/science.191908 191908 191908

[B3] Gavras H Brunner HR Turini GA Kershaw GR Tifft CP Guttelod S Gavras I Ukovish RA McKinstry DN New Engl J Med 1978 298 991 10.1056/NEJM197805042981803 205788 205788

[B4] Bravo EL Tarazi RC Hypertension 1979 1 39 232490 232490 10.1161/01.hyp.1.1.39

[B5] Brunner HR Gavras H Waebar B Kershaw GR Turini GA Vukovish RA McKinstry DN Ann Intern Med 1979 90 19 217289 217289 10.7326/0003-4819-90-1-19

[B6] Testa MA Anderson RB Nackley JF Hollenberg NK New Engl J Med 1993 328 907 10.1056/NEJM199304013281302 8446137 8446137

[B7] Antonaccio MJ Ann Rev Pharmacol Toxicol 1982 22 57 10.1146/annurev.pa.22.040182.000421 6282189

[B8] Komai T Ikeda T Kawai K Kameyama E Shendo H J Pharmacobio-Dynam 1981 4 677 10.1248/bpb1978.4.677 7038090

[B9] Seta Y Higuchi F Kawahara Y Nishimura K Okada R Int J Pharm 1988 41 245 10.1016/0378-5173(88)90201-3

[B10] Anaizi NH Swenson C Am J Hosp Pharm 1993 50 486 8442468 8442468

[B11] Horovitz SP Angiotensin Converting Enzyme Inhibitors, Mechanisms of Action and Clinical Implications: Procceedings of the A. N. Richards Symposium Sponsored by the Physiological Society of Philadelphia 1981 Urban & Schwarzenberg, Baltimore-Munich

[B12] Miazaki N Shionoiri H Uneda S Uneda G Yasuda G Gotoh E Fujishima S Kaneko Y Kawahara Y Yamazaki Y Nippon Jinzo Gakkai Shi 1982 24 421 6750186

[B13] Migdalof BH Wong KK Lan SJ Kripalani KJ Singhvi SM Fed Proc 1980 39 757

[B14] Drost JD Reier GE Jain NB U.S. Patent 4756911 1988

[B15] Guittard GV Carpenter HA Quan ES Wong PS Hamel LG US patent 5178867 1993

[B16] Nahata MC Morosco RS Hipple TF Am J Hosp Pharm 1994 51 95 8135269 8135269

[B17] Joshi YM Bachman WR Jain NB European Patent EP 288732 A2 1988

[B18] Thakur AB Jain NB U.S. Patent 4738850 1988

[B19] AprRashid A British Patent Application 2230441A 1990

[B20] Singh J Robinson DH Drug Dev Ind Pharm 1988 14 545 10.3109/03639048809151883

[B21] Matharu RS Singhavi NM Drug Dev Ind Pharm 1992 18 1567 10.3109/03639049209040859

[B22] DeCrosta MT Jain NB Rudnic EM U.S. Patent 4666705 1987

[B23] Sposito G Skipper NT Sutton R Park SH Soper AK Greathouse JA Proc Natl Acad Sci 1999 96 3358 10.1073/pnas.96.7.3358 10097044 10097044 PMC34275

[B24] Bergaya F Theng BKG Lagaly G Handbook of clay science 2006 Elsevier publication, Amsterdam

[B25] Ghanshyam VJ Hasmukh AP Bhavesh DK Hari CB Appl Clay Sci 2009 45 248 10.1016/j.clay.2009.06.001

[B26] Peppas NA Sahlin JJ Int J Pharm 1989 57 169 10.1016/0378-5173(89)90306-2

[B27] Reed-Hill RE Abbaschain R Physical metallurgy principles 1994 3 PWS publishing Company, Boston

[B28] Suguna Lakshmi M Sriranjani M Bava Bakrudeen H Suresh Kannan A Mandal AB Reddy Boreddy SR Appl Clay Sci 2010 48 589 10.1016/j.clay.2010.03.008

[B29] Pradhan R Budhathoki U Thapa P J Sci Eng Technol 2008 1 55

[B30] Korsmeyer RW Gurny R Doelker E Buri P Peppas NA Int J Pharm 1983 15 25 10.1016/0378-5173(83)90064-9 6644570

